# Decomposing rural-urban gap in unsafe disposal practice of child stool in India using nationwide sample survey data

**DOI:** 10.1038/s41598-024-56715-w

**Published:** 2024-03-19

**Authors:** Avijit Roy, Margubur Rahaman, Pradip Chouhan

**Affiliations:** 1 Department of Geography, State Aided College Teacher, Malda College, Malda, West Bengal 732101 India; 2 Doctoral Fellow, International Institute for Population Sciences (IIPS), Govandi Station Road, Deonar, Mumbai 400088 India; 3 Department of Geography, University of Gour Banga, Malda, West Bengal 732103 India

**Keywords:** Diseases, Risk factors

## Abstract

A significant rural–urban disparity in unsafe child stool disposal practices exists in India, yet existing research falls short in identifying the contributing factors to this gap. This study addresses the research gap by contextualizing the rural–urban divide in unsafe child stool disposal using data from the fifth round of the National Family Health Survey (NFHS-5, 2019–21). In particular, the study examines the prevalence and predictors of unsafe disposal practices, exploring associated contributing factors to this gap. The study involves a sample of 78,074 women aged 15–49 with a living child under 2 years, without any missing data related to the study interest. Employing descriptive statistics, the Pearson chi-square test, multilevel logistic regression, and the Fairlie decomposition model, the research aims to fulfill its objectives. The rural–urban gap in unsafe child stool disposal practices among the study participants was 22.3 percentage points (pp), with a more pronounced gap among the Scheduled Tribes (ST). Notably, the gap was particularly wide in Madhya Pradesh (33.9 pp), Telangana (27.5 pp), Gujarat (26.1 pp), and Rajasthan (25.8 pp). Predictors such as mother’s education, mass media exposure, household wealth quintile, and sanitation facilities proved significant irrespective of residence. However, religion, social group, and water facility on household premises emerged as significant factors in rural areas only. The study identified that 67% of the explained gap in unsafe child stool disposal practices was attributed to the rural–urban difference in household wealth. Other noteworthy contributors were ‘household sanitation facility’ (21.3%), ‘mother’s education level’ (3.9%), and ‘water facility on household premises’ (3.9%). These findings underscore the need for population and area-specific policy interventions, especially for individuals from socio-economically disadvantaged backgrounds, those with lower education levels, and limited exposure to mass media, particularly in states with a high prevalence of unsafe disposal practices. Such interventions are crucial to mitigating the existing rural–urban gap in unsafe child stool disposal practices.

## Introduction

While commendable progress has been observed in the use of improved sanitation facilities among adults in India, a noteworthy public health concern persists regarding the unsafe disposal practice of young child stool^1^. The latest National Family Health Survey (NFHS), conducted from 2019 to 21, highlights that despite 61% of households in India using improved toilet facilities, only 36% followed safe disposal practices for their child's stool^2^. Therefore, these statistics suggest that existing sanitation programs have predominantly focused on enhancing sanitation coverage and practices among adults, inadvertently overlooking the safe disposal practices of child stool^3^. Safe disposal practices for child stool include placing or rinsing the child’s stool in a toilet or latrine, burying it, or having the child use a toilet or latrine^2,4,5^. In contrast, unsafe disposal practices of child stool involve depositing or rinsing them into a drain, ditch, bush, garbage heap, or leaving them on the ground^5^. A recent study covering 34 low- and middle-income countries (LMICs) showed that half of households practiced unsafe disposal of child stool in this region^6^. The prevalence of unsafe disposal practices of child stool was found to be considerably high in India (64%), exceeding the LMICs average (50.6%). Although the prevalence of unsafe disposal practices for child stool remains high in India, there has been a considerable decline from 79% in 2005–06^4^ to 64% in 2019–20^5^. However, the recent temporal changes in unsafe disposal practices of child stool have remained stagnant at 66%–64% from 2015–16 to 2019–21 in India, warranting research attention^2,5^.

Existing research suggests that the unsafe disposal of child stool is a significant source of fecal exposure within household environments, positively linked with multiple health risks, particularly among children^6–9^. Children, due to their active engagement such as crawling and playing with their environment, are more susceptible to fecal exposure^8,9^. In particular, a positive association between unsafe disposal of child stool and diarrheal diseases among children has been found in India^4,10^ and other regions^11–14^. The likelihood of diarrhea was 11% higher among children whose feces were disposed of unsafely compared to those whose feces were disposed of safely in India^4^. Similarly, the unsafe disposal of child stool is a significant contributing factor to stunting and mortality among under-five children in India^5^ and elsewhere^6,15^. The prevalence of these adverse outcomes was notably higher in households practicing open defecation and unsafe disposal of a child's feces in India^4,5,10^.

In light of aforementioned findings^2,4,5,10^, a research investigation is relevant to contextualize the predictors of unsafe disposal practice of child stool in India. A substantial number of studies have explored the predictors of unsafe disposal practice of child stool and the associated negative impacts on child health and mortality in India^4,5,10^ and elsewhere^9,11–14,16^ at the national level, without dissecting the place of residence (rural vs. urban). Several socioeconomic and demographic factors have been identified as significant predictors of unsafe disposal practices of child stool in India and other regions^4,5,12,16^. In the context of Indian settings, significant predictors of unsafe disposal practices include the household wealth index, maternal age, maternal educational status, place of residence, and access to sanitation facilities^4,5,10^. Beyond these predictors, a prevailing perception in many societies persists that the feces of newborns and young children are harmless and not dirty, perpetuating the neglect of safe disposal practices for children's feces^7,11^.

Although previous studies consistently reported significantly higher odds of unsafe disposal practices of child stool in rural India compared to urban counterparts^4,5^, none have investigated the differences in predictors between rural and urban settings. Additionally, no previous studies have explored the contributing factors to the rural–urban gap in the unsafe disposal of children's feces. In India, the rural population is almost three times higher than the urban population, with substantial gaps in improved sanitation coverage and significant differences in socio-demographic and cultural backgrounds^2,17^. This could contribute to differentiating the prevalence and predictors of unsafe disposal of child stool between rural and urban areas in the country. Therefore, the present study aims to examine the rural–urban gap in the prevalence of unsafe disposal practice of child stool and its associated contributing factors. The study also explores place of residence-specific predictors of unsafe disposal practice of child stool, incorporating geographical variability. The findings from this research can inform the development of targeted, group-based policies and programs to promote the safe disposal of children's feces and minimize the negative impact of unsafe disposal practices.

## Material and methods

### Data source

The present study utilized secondary data from the fifth round of the NFHS, conducted during 2019–2021. The NFHS-5 survey collected comprehensive data on reproductive and child health, public health, family planning, and others^2^. The NFHS-5 sample was strategically designed to offer national, state/union territory (UT), and district-level estimates for various population and health-related indicators crucial for monitoring the Sustainable Development Goals (SDGs)^2^. However, certain indicators, such as sexual knowledge and behavior, domestic violence, and men's health indicators, are designed at the state/UT and national levels.Furthermore, NFHS-5 also included several indicators, including vital events, preschool education, and child stool disposal^2^.

### Sampling design

NFHS-5 used a two-stage stratified sampling technique to select a representative sample of households for data collection^2^. In the first sampling stage, the country was divided into geographical units known as Primary Sampling Units (PSUs). These PSUs were selected using probability proportional to size (PPS) sampling, where the size of the PSU was based on the population. The PSUs were selected from rural and urban areas separately to ensure proper representation of both settings. Within each rural stratum, PSUs were selected based on the literacy rate of women aged 6 + years. Within each urban stratum, a sample of Census Enumeration Blocks (CEBs) was selected as PSUs based on the scheduled castes and scheduled tribes (SC/ST) population. In the second stage, a complete listing of households was carried out in the selected PSUs. A fixed number of 22 households per cluster were selected with an equal probability of systematic selection from a newly created list of households in the selected PSUs. From the household listing, a systematic random sample of households was selected^2^. This involved selecting a starting point at random and then choosing every “k-th” household from the list, where “k” was a predetermined interval. In all, 30,456 Primary Sampling Units (PSUs) were selected across the country in NFHS-5 drawn from 707 districts, of which fieldwork was completed in 30,198 PSUs. Within each selected household, specific individuals were identified for various interviews. A random selection process was used to identify eligible respondents within the household. After the sample was collected, it was weighted to the data to adjust for any discrepancies between the sample and the larger population. This ensures that the survey results accurately reflect the characteristics of the entire population.The details of the respondent selection strategy and sampling weights are available in the NFHS report^2^. NFHS-5 utilizes four survey instruments to encompass a range of health and well-being concerns. These instruments comprise various questionnaires linked to general households, male health, female health, along with clinical, anthropometric, and biomedical (CAB) assessments^2^. In NFHS-5 (2019–21), a total of 653,144 occupied households were selected for the sample, of which 636,699 were successfully interviewed, with a 98% response rate^2^. A total of 747,176 eligible women between the ages of 15 and 49 years were identified in the interviewed households, and 724,115 of them were successfully interviewed with a 97% response rate.

### Study sample and participants

**Figure 1:**
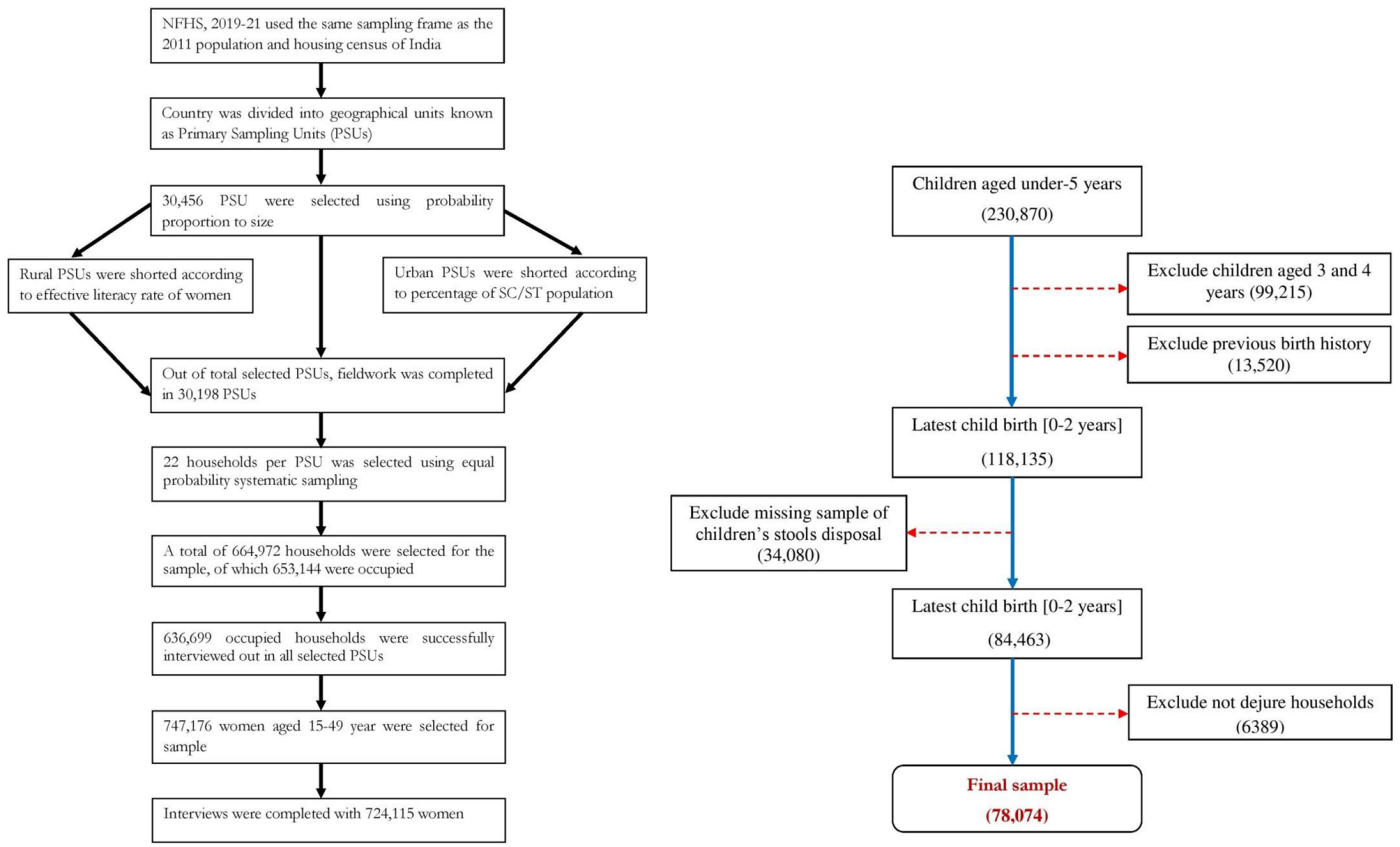
Schematic diagram for survey population and study sample.

Out of the total sample of 724,115 women aged 15–49 in NFHS-5, the present study selected a total of 78,074 women aged 15–49 with a living child under 2 years of age. A similar sample size was also chosen in the latest NFHS-5 report to depict the socio-demographic and regional variations in stool disposal practices in India^2^. A detailed description of the sample selection is presented in Fig. 1.

### Study variables

#### Outcome variable

The outcome variable was disposal practice of children's stools. This variable exhibits a dichotomous nature: safe disposal and unsafe disposal.Safe disposal practice of children's stools is considered when the child uses a toilet or latrine, when the fecal matter is placed or rinsed into a toilet or latrine, or when it is buried^2^. In contrast, unsafe disposal practices involve leaving fecal matter in open areas, disposing of it in garbage bins, rinsing it into drains, or employing alternative methods.The sub-categories of the outcome variable correspond to the latest NFHS-5 report, prepared by the International Institute for Population Sciences (IIPS) and the Inter-City Fund (ICF)^2^.

### Explanatory variables

In line with previous studies in India and elsewhere^10,11^, the present study selected a set of explanatory variables, including respondent’s age and education level, religion, social group, place of residence, household wealth quintile, mass media exposure, drinking water facility on household premises, household sanitation facility, *and region. A detailed description of explanatory variables is presented in *Supplementary Table 1*.*

### Statistical methods

Descriptive statistics (percentage distribution with 95% confidence interval) were used to describe the background characteristics of the study participants. A bivariate analysis was used to present the prevalence of unsafe disposal practice of children’s stoolsby selected explanatory variables. In addition, Pearson chi-square test was appliedto examinethe significance level of the bivariate associations. All descriptive results based on weighted sample using NFHS sampling weight. Sampling weights are employed to manage and regulate the proportional contribution of each participating unit, ensuring an accurate representation in the overall population estimate. The details of the sampling weight available in NFHS report^2^. The present study also calculated the absolute rural–urban gap in unsafe disposal practices of children's stools, considering selected background characteristics of the study participants. Further, two separate multilevel logistic regression analyses wereperformed to identify the significant predictors of unsafe disposal practice of child stool and geographical variation in the predictors. It's noteworthy that the NFHS dataset follows a hierarchical structure, with respondents nested within households (HH), households nested within primary sampling units (PSU), and these PSUs further nested within districts^2^. A random intercept logistic regression model was used for the current investigation^19,20^. The model has been selected for the likelihood of a mother withchild aged 0–24 months (*i*) in the HH *j*, PSU *k*, and district *l*practiced unsafe disposal of child stool (Ƴ_*ijkl*_ = 1) in rural areas, and same model replaced to study participants in urban areas.

$$logit\left({\pi }_{ijlk}\right)={\beta }_{o}+{BX}_{ijkl}+({f}_{0k}+{m}_{0jk}+{p}_{0jkl}+{s}_{0ijkl})$$

This model calculates the log odds of ${\pi }_{ijlk}$ adjusted for vector ${X}_{ijkl}$ of predictor variables assessed at the individual level. The parameter ${\beta }_{o}$ indicates the reference category of all variables with log odds of the unsafe disposal of child stool. The random effect within the parentheses is measured as a residual differential for the district *l* (*f*_*0l*_), PSU *k* (*m*_*0kl*_), HH *j* (*p*_*0jkl*_), and individual *i* (*s*_*0ijlk*_) considered to be independent and normally distributed with mean 0 and variance ${\sigma }_{f0}^{2}$, ${\sigma }_{m0}^{2}$, ${\sigma }_{p0}^{2}$, and ${\sigma }_{s0}^{2}$, respectively. The variances quantify between districts, between PSU, and between households, respectively, in the log-odds of unsafe disposal. The multilevel model results were presented in adjusted odds ratio (AOR) with 95% confidence interval (CI). Finally, the present study performed the Fairle decomposition model to contextualize the rural and urban gap in the unsafe disposal of children's stools. The present study utilized a binary-model-appropriate version of the Blinder-Oaxaca approach, developed by Fairlie^21^, to decompose the rural and urbangapin the unsafe disposal of children's stools.

The decomposition for a non-linear equation *y* = (*x*β) can be written as:

$$ \overline{y}^{o} - \overline{y}^{s} = { }\left[ {\mathop \sum \limits_{i = 1}^{{N^{o} }} \frac{{F\left( {x_{i}^{o} \beta^{o} } \right)}}{{N^{o} }} - \mathop \sum \limits_{i = 1}^{{N^{s} }} \frac{{F\left( {x_{i}^{s} \beta^{o} } \right)}}{{N^{s} }}} \right] + \left[ {\mathop \sum \limits_{i = 1}^{{N^{o} }} \frac{{F\left( {x_{i}^{s} \beta^{o} } \right)}}{{N^{s} }} - \mathop \sum \limits_{i = 1}^{{N^{s} }} \frac{{F\left( {x_{i}^{s} \beta^{s} } \right)}}{{N^{s} }}} \right], $$

where N^*J*^ is the sample size for interest group *j*. *y*^*j*^is the average probability of the binary outcome of the interest group *j,* and *F* is the cumulative distribution function from the logistic distribution. Here, superscripts *O* and *S* stand for rural and urban. The first term in brackets in the equation above represents the part of the gap between groups due to group differences in distributions of the entire set of independent variables, and the second term represents the part due to differences in the group processes determining levels of *y.* Explanatory variables underwent multicollinearity testing using variance inflation factors (VIF) before the decomposition analysis. Importantly, no evidence of multicollinearity was found (VIF < 2). Details of the Fairlie decomposition model available in previous literatures^21,22^. All statistical analyses were performed on STATA 12 SE software (Stata Corporation, College Station, Texas, USA).

### Ethics approval and consent to participate

This study is based on secondary data which is available in the public domain. Therefore, ethical approval is not required to conduct this study.

## Results

### Background characteristics of the study participants

Table 1 presents the background characteristics of the study population by place of residence. Most of the mothers were aged between 20 and 29 years in both rural and urban areas. Higher-educated mothers were more than two-fold higher in urban settings than their rural counterparts (30.4% vs. 12.6%). Most of the respondents were Hindu and belonged to the Other Backward Class (OBC), irrespective of the place of residence. However, the percentage of respondents belonged to ST social category was relatively low in urban settings compared to rural counterparts (4.4% vs. 12.7%). The percentage of the poorest households was noticeably higher in rural areas than in urban counterparts (30.8% vs. 4.2%). The respondents with no exposure to the media were nearly three-fold percentage points higher in rural areas (34.3%) than in urban (12%) counterparts. About 30% of households in rural areas had no water facility on the premises, whereas in urban areas, it was 17.9%. Open defecation practice was prevalent in rural areas (29.2%) than in urban (7.3%) counterparts. A detailed tabular presentation of study participants by type of disposal practice and place of residence is presented in the Supplementary Table 2.

**Table 1:** Background characteristics of the study participants by place of residence, India, National Family Health Survey, 2019–21.

Background characteristics	Rural			Urban		
	Weighted sample	Weighted percentage	95% CI	Weighted sample	Weighted percentage	95% CI
Mother’s age (years)						
15–19	3368	6.0	5.7, 6.0	646	3.1	2.9, 3.4
20–24	23,498	41.0	40.5, 41.3	6435	31.3	30.7, 31.9
25–29	20,373	35.0	35.0, 35.8	8120	39.5	38.8, 40.2
≥ 30	10,274	18.0	17.6, 18.2	5362	26.1	25.5, 26.7
Mother’s education						
No education	12,766	22.2	21.9, 22.5	2122	10.3	9.9, 10.7
Primary	7030	12.2	12.0, 12.5	1741	8.5	8.1, 8.8
Secondary	30,466	53.0	52.6, 53.4	10,439	50.8	50.1, 51.5
Higher	7251	12.6	12.3, 12.9	6260	30.4	29.8, 31.1
Religion						
Hindu	46,764	81.3	81.0, 81.6	15,024	73.1	72.5, 73.7
Muslim	8228	14.3	14.0, 14.6	4634	22.5	22.0, 23.1
Christian	1185	2.1	1.9, 2.2	449	2.2	2.0, 2.4
Others	1335	2.3	2.2, 2.4	455	2.2	2.0, 2.4
Social group						
GEN	8443	14.7	14.4, 15.0	5319	25.9	25.3, 26.5
SC	13,820	24.0	23.7, 24.4	4129	20.1	19.5, 20.6
ST	7315	12.7	12.4, 13.0	911	4.4	4.1, 4.7
OBC	24,819	43.2	42.7, 43.6	8936	43.5	42.8, 44.1
Don't know	3114	5.4	5.2, 5.6	1267	6.2	5.8, 6.5
Household wealth quintile						
Poorest	17,715	30.8	30.4, 31.2	863	4.2	3.9, 4.5
Poorer	14,760	25.7	25.3, 26.0	1822	8.9	8.5, 9.3
Middle	11,980	20.8	20.5, 1.2	3478	16.9	16.4, 17.4
Richer	8735	15.2	14.9, 15.5	5899	28.7	28.1, 29.3
Richest	4322	7.5	7.3, 7.7	8499	41.3	40.7, 42.0
Mass media exposure						
No	19,739	34.3	33.9, 34.7	2469	12.0	11.6, 12.5
Partial	34,678	60.3	59.9, 60.7	16,042	78.0	77.4, 78.6
High	3096	5.4	5.2, 5.6	2052	10.0	9.6, 10.4
Water facility onhousehold premises						
Yes	53,446	69.4	69.0, 69.8	19,646	82.1	81.6, 82.6
No	3062	30.6	30.2, 30.9	288	17.9	17.4, 18.4
Household sanitation facility						
Improved	39,075	68.5	68.1, 68.9	18,505	90.3	89.9, 90.7
Unimproved	1324	2.3	2.2, 2.4	480	2.3	2.1, 2.5
Open defecation	16,626	29.2	28.8, 29.5	1504	7.3	7.0, 7.7
Region						
North	7140	12.4	12.1, 12.7	3303	16.1	15.6, 16.6
Central	17,192	29.9	29.5, 30.3	4456	21.7	21.1, 22.2
East	17,000	29.6	29.2, 29.9	3422	16.6	16.1, 17.2
Northeast	2530	4.4	4.2, 4.6	443	2.2	2.0, 2.4
West	5833	10.1	9.9, 10.4	3869	18.8	18.3, 19.4
South	717	13.6	13.3, 13.9	5069	24.7	24.1, 25.2
**Total**	57,512	100	–	20,562	100	–

*CI* Confidence interval.

### Rural–urban gap in prevalence of unsafe disposal practice of child stool

In the study population, the rural–urban gap in the prevalence of unsafe disposal of child stoolwas 22.3 percentage points (pp) at the national level (Table 2). Similarly, the gap also varied across different demographic and socioeconomic backgrounds. For instance, the rural–urban gap in the unsafe disposal practice of child stool was substantially higher among mothers aged 30 years and above (26.6 pp) than their counterparts. Among mothers with no education, the rural–urban gap in the unsafe disposal practice of child stool was about 20.4 pp. Similar results were also observed among mothers with primary and secondary education levels, with the gap ranging between 20.9 pp and 19.8 pp. The rural–urban gap in the unsafe disposal practiceof child stoolwas higher among the Hindus (23.9 pp), followed by the Muslims (18.1 pp). The unsafe disposal practiceof child stool was considerably higher among the ST individuals than their counterparts in rural areas, indicating a higher rural–urban gap (28.5 pp). The rural–urban gap in the unsafe disposal practice of child stool varied across the household wealth quintiles (range: 3.0 pp to 7.1 pp). In particular, the prevalence of unsafe disposal practice of child stool was more than double in households with the poorest wealth quintile than in the richest counterparts, irrespective of the place of residence. The unsafe disposal practiceof child stoolwas predominantly higher among mothers who had no mass media exposure (76.8%), belonged to households with no water facility on the premises (74.3%), and had unimproved sanitation facilities (70.7%) in rural areas than their urban counterparts. The rural–urban gap in the unsafe disposal practice of child stool was highest in the central region (24 pp), while the gap was relatively low in the north (10.8 pp) region.

**Table 2:** Rural–urban difference in prevalence of unsafe disposal practice of child stool by selected background characteristics among study participants, India, National Family Health Survey, 2019–21.

Background characteristics	Rural			Urban			Rural–urban absolute difference (in percentage points)
	Prevalence (%)	95% CI	Chi-square p-value	Prevalence (%)	95% CI	Chi-square p-value	
Mother’s age (years)							
15–19	73.0	71.5, 74.5		54.0	50.1, 57.8		19.0
20–24	68.1	67.5, 68.7	≤ 0.001	49.4	48.2, 50.7	≤ 0.001	18.7
25–29	65.5	64.8, 66.1		42.8	41.7, 43.9		22.7
≥ 30	67.2	66.2, 68.1		40.6	39.3, 41.9		26.6
Mother’s education							
No education	78.3	77.6, 79.0		57.9	55.8, 60.0		20.4
Primary	73.4	72.4, 74.4	≤ 0.001	52.5	50.1, 54.8	≤ 0.001	20.9
Secondary	64.9	64.3, 65.4		45.1	44.1, 46.0		19.8
Higher	52.2	51.0, 53.3		37.3	36.0, 38.4		14.9
Religion							
Hindu	69.0	68.6, 69.4		45.1	44.3, 45.8		23.9
Muslim	61.8	60.8, 62.9	≤ 0.001	43.7	42.3, 45.1	≤ 0.001	18.1
Christian	62.1	59.3, 64.9		51.0	46.4, 55.6		11.1
Others	46.4	43.8, 49.1		34.8	30.4, 39.1		11.6
Social group							
GEN	55.6	54.5, 56.6		39.6	38.3, 40.9		16.0
SC	69.5	68.7, 70.2		52.0	50.5, 53.5		17.5
ST	78.2	77.2, 79.1	≤ 0.010	49.7	46.5, 53.0	0.032	28.5
OBC	67.4	66.8, 68.0		45.0	43.9, 46.0		22.4
Don't know	63.0	61.3, 64.7		35.9	33.2, 38.5		27.1
Household wealth quintile							
Poorest	82.7	82.2, 83.3		79.6	76.9, 82.3		3.1
Poorer	72.1	71.3, 72.8		65.7	63.6, 67.9		6.4
Middle	62.4	61.5, 63.3	≤ 0.001	55.3	53.7, 57.0	≤ 0.001	7.1
Richer	50.1	49.0, 51.1		43.2	41.9, 44.5		6.9
Richest	36.2	34.8, 37.6		33.2	32.2, 34.2		3.0
Mass media exposure							
No	76.8	76.2, 77.3		57.6	55.6, 59.5		19.2
Partial	63.4	62.9, 63.9	0.004	43.3	42.6, 44.1	0.002	20.1
High	51.0	49.2, 52.8		39.4	37.3, 41.5		11.6
Water facility on premises							
Yes	64.2	63.8, 64.7		42.8	42.1, 43.6		21.4
No	74.3	73.6, 74.9	0.031	53.1	51.4, 54.7	≤ 0.001	21.2
Sanitation facility							
Improved	59.1	58.6, 59.6		41.1	40.3, 41.8		18.0
Unimproved	70.7	68.3, 73.2	≤ 0.001	56.4	51.9, 60.8	≤ 0.001	14.3
Open defecation	86.2	85.7, 86.7		83.8	82.0, 85.7		2.4
Region							
North	51.1	49.9, 52.3		40.3	38.6, 41.9		10.8
Central	69.6	68.9, 70.3		45.6	44.2, 47.1		24.0
East	75.8	75.1, 76.4		55.1	53.4, 56.7		20.7
Northeast	73.9	72.2, 75.6	≤ 0.001	59.7	55.2, 64.3	≤ 0.001	14.2
West	60.1	58.9, 61.4		36.9	35.4, 38.4		23.2
South	61.8	60.7, 62.9		44.2	42.9, 45.6		17.6
Total	67.6	67.2, 67.9		45.3	44.7, 46.0		22.3

All percentages are weighted.

CI = Confidence interval.

### State level patterns of rural–urban gap in unsafe disposal practice of child stool

**Figure 2:**
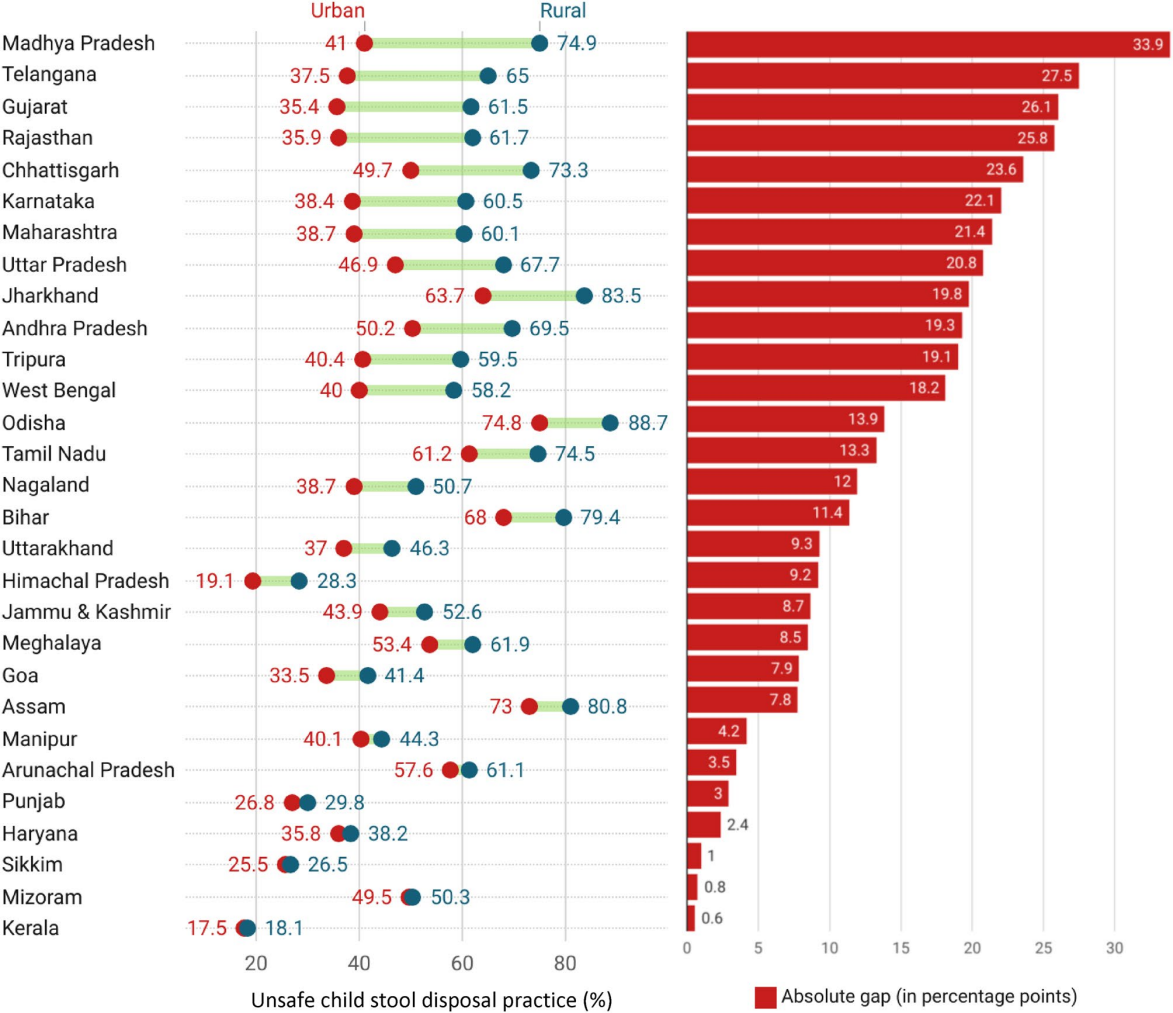
Rural–urban gap in unsafe disposal practice of child stool in India, NFHS (2019–21). Source: Author's calculation based on NFHS-5 (2019-21) data .

Figure 2 presents the rural–urban gap in unsafe disposal practice of child stool across the states of India. The rural–urban gap varied substantially across states with highest being Madhya Pradesh (33.9 pp) and followed by Telangana (27.5 pp), Gujarat (26.1 pp) and Rajasthan (25.8 pp). Conversely, the rural–urban gap in unsafe disposal of child stool was less prominent in Kerala (0.6 pp), Mizoram (0.0.8 pp), and Sikkim (1.0 pp).

### Rural–urban difference in predictors of unsafe disposal practice of child stool

The present study reveals a notable rural–urban variation in the predictors of unsafe disposal practice of child stool (Table 3). For instance, mother’s education, household wealth quintile, mass media exposure, sanitation, and region were identified as significant predictors of unsafe disposal practiceof child stool in both rural and urban areas. In contrast, mother’s age, the water facility on household premises, religion, and social group were found to be significant predictors of unsafe disposal practice of child stool in rural areas only. Similarly, although geographical variance in predictors of unsafe disposal practice of child stool was found to be highest at the household level in both rural and urban areas, the level of variance was slightly higher in urban areas (4.42; 95% CI: 2.76–7.06) than in rural areas (4.00; 95% CI: 3.05–5.24) (Table 3). Increasing levels of mother's education, household wealth quintile, and mass-media exposure associate with a decreased probability of unsafe disposal practiceof child stool. In particular, mothers with higher education in rural areas had 42% (AOR:0.58; 95% CI: 0.50–0.67) lower adjusted odds of unsafe disposal practice of child stool, while their urban counterparts showed a 36% (AOR:0.74; 95% CI: 0.55–1.00) decrease compared to those with no formal education. Similarly, mothers from the richest household quintile were 84% (AOR:0.16; 95% CI:0.12–0.20) and 87% (AOR:0.13; 95% CI: 0.08–0.23) less likely to practice unsafe disposal of child stool in rural and urban areas, respectively, compared to poorest counterparts. Those with high mass-media exposure exhibited 32% (AOR:0.68; 95% CI:0.58–0.81) and 30% (AOR:0.70; 95% CI:0.51–0.97) lower odds of unsafe disposal than reference category i.e., no mass media exposure in rural and urban areas, respectively. Notably, mothers practicing open defecation were 11.48 (AOR:11.48; 95% CI:6.95–18.95) and 4.31 (AOR:4.31; 95% CI:3.70–5.03) times more likely to engage in unsafe disposal than those with improved sanitation practices in urban and rural areas, respectively. However, a significant variation in adjusted odds in unsafe disposal practice of child stool was observed across religious and social groups observed in rural areas only. In particular, the mothers belonged to other and Muslim religious communities had 35% (AOR:0.65; 95% CI:0.51–0.83) and 16% (AOR:0.84; 95% CI:0.73–0.97) lower odds of unsafe disposal practiceof child stool, respectively, compared to Hindu counterparts. Concurrently, ST and scheduled caste (SC) exhibited 31% (AOR:1.31; 95% CI: 1.09–1.41) and 19% (AOR:1.19; 95% CI:1.06–1.30) higher odds of unsafe disposal practiceof child stool, respectively than the general population. Households lacking water facilities on premises had a 31% (AOR:1.31; 95% CI: 1.20–1.44) higher likelihood of unsafe disposal practice of child stool than their counterparts in rural areas. Regionally, central, east, and northeast areas displayed considerably higher adjusted odds of unsafe disposal practiceof child stool than their north region counterparts in both rural and urban areas. In rural areas, south and west regions exhibited significantly higher adjusted odds of unsafe disposal practiceof child stool compared to the north region.

**Table 3:** Adjusted odds ratio (AOR) of unsafe disposal practice of child stool by background characteristics among study participants, India, National Family Health Survey, 2019–21.

Background characteristics	Rural		Urban	
	AOR [95% CI]	p-value	AOR [95% CI]	p-value
Mother’s age (years)				
15–19	Reference		Reference	
20–24	0.77 [0.65, 0.90]	0.010	0.98 [0.65, 1.47]	0.081
25–29	0.67 [0.57, 0.80]	≤ 0.001	0.77 [0.51, 1.17]	0.079
≥ 30	0.66 [0.55, 0.79]	≤ 0.001	0.67 [0.44, 1.02]	0.070
Mother’s education				
No education	Reference		Reference	
Primary	0.97 [0.85, 1.10]	0.970	1.01 [0.74, 1.38]	0.110
Secondary	0.75 [0.67, 0.83]	≤ 0.001	0.87 [0.67, 1.12]	0.170
Higher	0.58 [0.50, 0.67]	≤ 0.001	0.74 [0.55, 1.00]	0.031
Religion				
Hindu	Reference		Reference	
Muslim	0.84 [0.73, 0.97]	0.050	0.88 [0.71, 1.09]	0.080
Christian	0.73 [0.57, 0.94]	0.041	0.78 [0.50, 1.22]	0.071
Others	0.65 [0.51, 0.83]	≤ 0.001	0.86 [0.56, 1.32]	0.066
Social group				
GEN	Reference		Reference	
SC	1.19 [1.06, 1.30]	0.010	1.06 [0.87, 1.29]	0.060
ST	1.31 [1.09, 1.41]	0.010	1.25 [0.99, 1.58]	0.057
OBC	0.98 [0.88, 1.09]	0.980	1.05 [0.74, 1.47]	0.090
Don't know	0.92 [0.75, 1.13]	0.920	0.85 [0.59, 1.22]	0.064
Household wealth quintile				
Poorest	Reference		Reference	
Poorer	0.67 [0.60, 0.74]	≤ 0.001	0.44 [0.28, 0.69]	≤ 0.001
Middle	0.43 [0.38, 0.50]	≤ 0.001	0.35 [0.22, 0.55]	≤ 0.001
Richer	0.26 [0.22, 0.31]	≤ 0.001	0.21 [0.13, 0.35]	≤ 0.001
Richest	0.16 [0.12, 0.20]	≤ 0.001	0.13 [0.08, 0.23]	≤ 0.001
Mass media exposure				
No	Reference		Reference	
Partial	0.93 [0.85, 1.01]	0.530	0.89 [0.71, 1.12]	0.190
High	0.68 [0.58, 0.81]	≤ 0.001	0.70 [0.51, 0.97]	0.035
Water facility on premises				
Yes	Reference		Reference	
No	1.31 [1.20, 1.44]	≤ 0.001	0.86 [0.71, 1.06]	0.116
Household sanitation facility				
Improved	Reference		Reference	
Unimproved	1.19 [1.00, 1.42]	0.031	1.24 [0.82, 1.90]	0.071
Open defecation	4.31 [3.70, 5.03]	≤ 0.001	11.48 [6.95, 18.95]	≤ 0.001
Region				
North	Reference		Reference	
Central	3.58 [2.58, 4.98]	≤ 0.001	2.02 [1.32, 3.08]	0.010
East	7.34 [5.04, 10.68]	≤ 0.001	4.62 [2.74, 7.79]	≤ 0.001
Northeast	2.42 [1.67, 3.50]	≤ 0.001	3.08 [1.78, 5.34]	≤ 0.001
West	1.86 [1.25, 2.75]	0.010	0.78 [0.49, 1.26]	0.780
South	2.85 [2.01, 4.05]	≤ 0.001	1.56 [1.02, 2.39]	0.027
Constant	3.72 [2.67, 5.18]	≤ 0.001	2.95 [1.46, 5.94]	≤ 0.001
Random intercept parameter				
Var (district)	1.37 [1.11, 1.69]		1.53 [1.07, 2.19]	
Var (PSU)	3.44 [2.90, 4.07]		3.35 [2.43, 4.62]	
Var (HHs)	4.00 [3.05, 5.24]		4.42 [2.76, 7.06]	
ICC (district) (%)	11.3		12.2	
ICC (PSU) (%)	39.8		38.8	
ICC (HHs) (%)	72.8		73.9	
Model fit statistics				
Wald test *χ*^2^	536.58	≤ 0.001	153.75	≤ 0.001
LR test vs. logistic regression	≤ 0.001		≤ 0.001	

*AOR* Adjusted odds ratio, *CI* Confidence interval.

### Findings from decomposition analysis

The rural–urban gap in unsafe disposal practice of child stool was about 21 percentage points, which explained 80% of the overall difference (Supplementary Table3). In particular, the proportion of unsafe disposal practice of child stool in rural areas was 0.66, considerably higher than in urban counterparts (0.45). Household wealth quintile was examined as a key contributor to the rural–urban difference in unsafe disposal practice of child stool, explaining about 67% of the variance (Table 4). Household sanitation facilities (21.3%), mother’s education (3.9%) and water facility on household premises (3.9%) were also identified as significant contributors to the rural–urban difference in unsafe disposal practice of child stool.

**Table 4:** Percentage contribution of selected each predictors to the rural–urban gap in the unsafe disposal practice of children stool among study participants, India, National Family Health Survey, 2019–21.

Predictors	Coefficient	p-value	95% Confidence interval	% Contribution
Mother’s age	0.0035	≤ 0.001	0.0026 to0.0044	2.2
Mother’s education	0.0065	≤ 0.001	0.0046 to0.0083	3.9
Religion	0.0006	≤ 0.001	0.0005 to0.0007	0.3
Social group	0.0007	≤ 0.001	0.0004 to0.0009	0.4
Household wealth quintile	0.1110	≤ 0.001	0.1059 to0.1160	66.9
Mass media exposure	0.0042	≤ 0.001	0.0024 to0.0061	2.5
Water facility on household premises	0.0065	≤ 0.001	0.0052 to0.0077	3.9
Household sanitation facility	0.0353	≤ 0.001	0.0336 to0.0370	21.3
Region	-0.0023	≤ 0.001	− 0.0030 to − 0.0016	-1.4

## Discussion

The present study aims to contribute to the scientific literature by examining the rural–urban variation in prevalence and predictors of unsafe disposal practices of child stool among study participants in India, using the latest nationally representative sample survey data, i.e., NFHS-5 (2019–21). The study also explores the contributing factors of the rural–urban gap in unsafe disposal practices of child stool among the study participants. Existing studies have shown that the prevalence of unsafe disposal practices of child stool is higher in rural areas than urban counterparts in India^4,5^, using the third and fourth rounds of NFHS survey. However, these studies are failed to answering what the contributing factors are in this respective rural–urban gap^4,5^. Subsequently, the existing studies scientifically predicted the significant predictors of unsafe disposal practices of child stool at the national context but overlooked to contextualize rural–urban variation in these predictors^4,5^. In addition, a considerable number of prior studies have delved into the predictors of unsafe child stool disposal practices at the rural level in India^23–25^. Nevertheless, these studies, being micro-level analyses, fall short in providing insights into the broader macro-level context^23–25^. Therefore, the present study findings will be helpful to the research community, academia, and public health interest groups to understand the rural–urban dichotomy in prevalence, predictors of unsafe disposal practices of child stool, and associated contributors in India from the perspective of selected study participants.

The first and foremost finding of this study reveals a significant rural–urban gap in the prevalence of unsafe disposal practices of child stool observed in India, aligning with consistent trends identified in prior research^4,5^. While earlier studies acknowledged this gap^4,5,23–25^, they inadequately explicated the key contributing factors to the rural–urban gap in unsafe child stool disposal practices. In contrast, the present study systematically identifies household wealth quintile, household sanitation facilities, water availability on household premises, and women's education as pivotal contributors to this specific rural–urban gap. The existing scientific literature emphasizes that issues such as household-level poverty, unimproved sanitation, or open defecation practices, along with the lack of household-level piped water connectivity and female illiteracy, are notably more prevalent in rural areas compared to their urban counterparts^2,17,22,26,27^. Consequently, the observed rural–urban differentials in unsafe child stool disposal practices may result of rural–urban gap in poor household wealth quintile, availability of improved sanitation facilities, access to water on household premises, and women's higher education. Beyond the quantifiable factors examined in this study, it is important to acknowledge the potential influence of numerous unobserved factors, including socio-cultural beliefs and practices, as well as an individual's health awareness^7,11,23,24,28^.

Furthermore, the current study highlights a notable rural–urban gap in the prevalence of unsafe child stool disposal in most of the Indian states, particularly evident in Madhya Pradesh, Telangana, Gujarat, and Rajasthan. Conversely, Kerala, Mizoram, Sikkim, and several other states exhibit no such gap between rural and urban areas. Previous research has consistently highlighted wide rural–urban gaps in water and sanitation facilities and socio-cultural indicators in states like Madhya Pradesh and Rajasthan, whereas such gaps are comparatively lower in Kerala^2,17,22,26,27^. Given this broader context, the rural–urban gap highlighted in our study may be influenced by these existing disparities. To comprehensively understand the reasons behind the state-level variations in the rural–urban gap in the prevalence of unsafe child stool disposal practices, further investigation is warranted.A state-wise analysis can provide valuable insights into the factors influencing this variation, making it crucial to consider states as the primary unit of study.

Secondly, household-level factors such as wealth quintile, sanitation, and water facilities were found to be significant predictors of child stool disposal practices among the study population. Consistent with earlier studies in India and elsewhere^2,4,5,12,23,24^, the study’s findings affirm that improving household wealth parallels a decline in unsafe disposal practices. Improved household prosperity facilitates better living conditions, including upgraded sanitation facilities. As a result, better living conditions promote safe disposal services and practices^29^. In line with previous studies^4,5,30^, the practice of unsafe disposal of child stool was found to be prevalent among households with unimproved sanitation facilities or open defecation practices in the current study. Households with unimproved sanitation facilities and open defecation practices have no option to place child stool at safe places, for this reason, they throw it in open spaces or other unsafe places^31,32^. Similar to previous studies^4,5,22,30–32^, water and improved sanitation facilities appeared as household factors influencing stool disposal practices. The current study explored that households lacking water facilities, especially in rural India, are prone to unsafe disposal. Prior research highlighted the significant role of water connectivity and improved sanitation facilities at the house for adult hygienic sanitation practices in India^22–25,30–32^; the current study expands its significance for safely disposing of children’s stools. It is crucial to consider these factors while formulating policies and interventions to promote safe disposal practices.

Thirdly, similar to previous studies in India^4,5,23–25,30^ and elsewhere^20,29^, individual-level factors such as mother’s education and mass media exposure play a positive role in safe disposal practices of child stool. Prior studies suggest that higher-educated and mass media-exposed mothers are more aware of the associated risks of unsafe children’s stool disposal, adopting safer behaviors and healthier lifestyles^33^. In Indian settings, mothers are the primary caregivers of children, and therefore, a significant linkage between mother’s education and child health has been observed in many previous studies^33^. For instance, the risk of malnutrition and mortality among under-five children is more common among children with lower-educated mothers than their higher-educated counterparts^34–36^.The current study found that mother’s age is an another individual-level factor of child stool disposal practices among the study participants, particularly in rural settings. In addition, the unsafe disposal practice of child stool is prevalent among teenage mothers in rural areas. Numerous scholarly investigations underscore the pressing issue of child marriage and early-age childbearing prevalent in rural India^37,38^. This societal concern predominantly affects individuals marked by low literacy levels and membership in economically disadvantaged households^37,38^. Consequently, mothers who undergo early-age childbearing, especially those with lower educational attainment and economic vulnerability^37^, may have limited awareness concerning the health risks linked to the unsafe disposal of child stool. Furthermore, the lack of improved sanitation facilities among child married women^38^, primarily due to household-level poverty, poses a significant barrier to following healthy lifestyle. It is crucial to consider these factors while formulating policies and interventions to promote safe disposal practices.

Fourthly, religious and social affiliations have emerged as substantial predictors of unsafe child stool disposal practices in the current study, specifically in rural areas. In particular, a higher likelihood of unsafe disposal was observed among mothers identifying with the Hindu religious group in rural areas, while no such significant risk was evident within the same religious group in urban areas. This highlights the nuanced influence of lifestyle over religion. The findings suggest that socio-cultural modernization may mitigate the impact of these factors on disposal practices in urban areas^39^. Despite the heterogeneity of urban spaces in terms of population and socio-cultural aspects, the rigidity of religious and caste affiliations weakens with modernity^39^. The current study also found that the probability of unsafe child stool disposal is higher among SC and ST communities compared to the general caste in rural areas, whereas it remained statistically insignificant in urban contexts. Numerous prior studies underscore the presence of social-group-based (caste-based) inequalities in household sanitation, water facilities, and socio-cultural prosperity, particularly pronounced in rural India as compared to urban counterparts^40^. In urban areas, disparities in living standards are primarily driven by individual socio-economic prosperity rather than caste identity.Although the association between social groups and child stool disposal practices holds significance in rural settings, it cannot be dismissed in the context of policy implications, given that nearly 70% of India's population resides in rural areas^17^.The conclusive finding of the current study suggests a slightly higher household-level variance in unsafe child stool disposal practices in urban areas compared to their rural counterparts. This elevated household-level variance in urban areas may be attributed to the more pronounced household-level inequality in sanitation practices within urban settings^22,41^. Notably, a significant number of urban residents utilize community toilets situated at a considerable distance from their households^41^, which may discourage the regular disposal of child stool in the toilet. Instead, it is common for urban residents to opt for easily accessible door-step dustbins.

### Strengths

The study has several some strengths. Foremost, the present study filled existing research gap by contextualizing rural–urban variation in the prevalence and predictors of unsafe child stool disposal practices in India. Furthermore, the study used nationally representative sample survey data, i.e., the NFHS, which minimized the risk of sampling errors in area-specific (rural vs. urban) study findings.

### Limitations

Notwithstanding its strengths, this study has certain limitations that warrant consideration. As a cross-sectional study, establishing a causal relationship between outcomes and independent variables remains challenging. Secondly, the reliance on self-reported data introduces the possibility of both social desirability bias and recall bias, compromising the absolute accuracy of the findings. Thirdly, it's important to note that the study's depiction of prevalence and predictors is based on the period of the survey and, at the present moment, inadvertently failed to capture the usual disposal practice of child stool. Fourthly, due to the quantitative data structure of NFHS, several qualitative nuances such as cultural norms, habits, and beliefs were missed in the study. Lastly, the present study overlooked the association between unsafe disposal practices and child health outcomes, therefore, recommends further study to investigate it.

## Conclusion

In conclusion, this study provides valuable insights into the rural–urban disparities in unsafe disposal practices of child stool among study population in India. The significant rural–urban gap underscores the importance of tailoring interventions to address specific contextual factors influencing child stool disposal practices. Household-level factors such as wealth quintile, sanitation facilities, water availability, and women's education have emerged as key contributors to this gap. Policies aimed at improving these factors in rural areas could substantially reduce unsafe disposal practices. Concurrently, policies promoting economic prosperity and enhancing sanitation infrastructure at the household level can significantly contribute to safer practices. Furthermore, emphasizing the role of maternal education and mass media exposure is crucial in fostering awareness and promoting healthier behaviors in child care. The state-level variations emphasize the need for targeted strategies, acknowledging the diverse socio-economic and cultural landscapes across different regions of India. States exhibiting wider rural–urban gaps, such as Madhya Pradesh and Rajasthan, require focused interventions to address existing disparities in water and sanitation facilities. On the other hand, states like Kerala, where the gap is minimal, can serve as models for successful policies that bridge rural–urban differentials. Religious and social affiliations were identified as significant predictors, particularly in rural areas. Understanding the nuanced influence of lifestyle over religion and the impact of socio-cultural modernization is vital for tailoring interventions that consider the evolving dynamics in urban areas.The study underscores the need for holistic policies addressing the rural–urban gap in child stool disposal practices, taking into account the multifaceted influences at the household, individual, and societal levels. By integrating these findings into public health interventions, policymakers can work towards fostering safer and healthier practices for child stool disposal, contributing to the overall well-being of the population.

### Supplementary Information

Supplementary Information.

## Data Availability

The dataset analyzed during the current study are available in the Demographic and Health Surveys (DHS) repository, https://dhsprogram.com/data/available-datasets.cfm.
