# Supplementary material for: Decomposing rural-urban gap in unsafe disposal practice of child stool in India using nationwide sample survey data
Source: Sci Rep. 2024 Mar 19;14:6632. doi: 10.1038/s41598-024-56715-w (PMC10950857; doi:10.1038/s41598-024-56715-w)
Supplement: Supplementary file 1 — Supplementary Information. [file 41598_2024_56715_MOESM1_ESM.docx]

Supplementary Table 1: Operational description of the predictor variables

| **Independent variables** | **Operational description** | **Coding** |
| --- | --- | --- |
| Mother’s age (years) | The age of the respondents is divided into 4 categories | 15-19 years (1), 20-24 years (2), 25-29 years (3), ≥30 years (4) |
| Mother’s education | Mother’s education is classified into four categories depending on years of schooling: No education = no years of schooling; primary = 1–5 years of schooling; secondary = 6–10 years of schooling; and above secondary = more than 10 years of schooling. | No education (0), Primary (1), Secondary (2), Higher(3) |
| Religion | In the survey, women were asked to specify their religion, with response options including Hindu, Muslim, Christian, Sikh, Bud-dhist/Neo Buddhist, Jain, Jew, Parsi/Zoroastrian, Others, and no religion. Respondents were categorized into four distinct groups:‘Hindu’, ‘Muslim’, ‘Christian’, ‘Others’. The ‘Others’ category encompasses individuals who identified as Sikh, Buddhist/Neo Buddhist, Jain, Jew, Parsi/Zoroastrian, specified ‘Others’, or indicated ‘no religion. | Hindu (1), Muslim (2), Christian (3), Others (4) |
| Social group | Social groups have been categorized into four distinct groups in accordance with the official classification of the Government of India: ‘General’: This broad category includes all other communities and castes that do not fall under the SC, ST, or OBC classifications. ‘Scheduled Caste’ (SC): These communities/castes, often referred to as Dalits, have historically experienced oppression and marginalization in India. ‘Scheduled Tribe’ (ST): This category encompasses indigenous and tribal communities who have historically faced social and economic disadvantages. ‘Other Backward Classes’ (OBC): These include social groups/castes have faced social and educational disadvantages, but they may not be part of the SC or ST. | General (1), SC (2), ST (3), OBC (4), Don’t know (5) |
| Household wealth quintile | The wealth index is a composite index of household amenities and assets; it indicates the socioeconomic condition; every household is given a score based on the number of consumer goods they own. A total of 33 assets and housing characteristics were considered for preparing a factor score using Principal Component Analysis. After that this factor score is divided into five equal categories ‘poorest’ (1); ‘poorer’ (2); ‘middle’(3); ‘richer’ (4); ‘richest’ (5), each with 20 % of the population. | Poorest (1), Poor (2), Middle (3), Rich (4),Richest (5) |
| Mass media exposure | Exposure to mass media was assessed from the frequency of reading newspapers/magazines, watching television, and listing radio. On the basis of these three media, respondents were categorized into three groups: low exposure (none of these media accessed), partial exposure (access to one or two media), and high exposure (access to all three mass media) | Low exposure (1), Partial exposure (2), High exposure (3) |
| Water facility at premises | Water facility is classified into two categories | Yes (1), No (2) |
| Sanitation facility | Sanitation facility is classified into three categories as per demographic health survey (DHS) guideline | Improved (1), Unimproved (2), No facility/open defecation (3) |
| Place of residence | Place of residence is classified into two categories | Urban (1), Rural (2) |
| Geographical region | India has been divided into 6 regions based on geographical and cultural settings. These are I. North region (Jammu and Kashmir, Himachal Pradesh, Punjab, Haryana, Chandigarh, Rajasthan, Delhi), II. Central region (Uttar Pradesh, Madhya Pradesh Chhattisgarh and Uttarakhand), III. East region (Bihar, Jharkhand, West Bengal and Orissa), IV. Northeast region (Arunachal Pradesh, Assam, Manipur, Meghalaya, Mizoram, Nagaland, Sikkim, and Tripura), V. West region (Gujarat, Maharashtra, Goa, Dadra Nagar Haveli and Daman Diu), VI. South region (Andhra Pradesh, Karnataka, Kerala, Tamil Nadu Puducherry, Telangana, Andaman Nicobar, Lakshadweep). | North (1), Central (2), East (3), Northeast (4) West (5), South (6), |

Supplementary Table 2: Study sample distribution by type of disposal practice for child stool and place of residence, India, National Family Health Survey, 2019-21

| **Background characteristics** | Rural | | | | Urban | | | |
| --- | --- | --- | --- | --- | --- | --- | --- | --- |
|  | Unsafe | | Safe | | Unsafe | | Safe | |
| **Mother’s age (years)** | N (weighted) | % | N (weighted) | % | N (weighted) | % | N (weighted) | % |
| 15-19 | 2,459 | 6.4 | 909 | 4.8 | 349 | 3.8 | 297 | 2.6 |
| 20-24 | 16,002 | 41.4 | 7496 | 39.9 | 3182 | 34.7 | 3253 | 28.6 |
| 25-29 | 13,343 | 34.5 | 7030 | 37.4 | 3476 | 37.9 | 4644 | 40.8 |
| ≥30 | 6,900 | 17.8 | 3374 | 17.9 | 2175 | 23.7 | 3187 | 28.0 |
| **Mother’s education** |  |  |  |  |  |  |  |  |
| No education | 9,998 | 25.8 | 2769 | 14.7 | 1228 | 13.4 | 894 | 7.9 |
| Primary | 5,160 | 13.3 | 1870 | 9.9 | 914 | 10.0 | 827 | 7.3 |
| Secondary | 19,762 | 51.1 | 10704 | 56.9 | 4707 | 51.3 | 5732 | 50.4 |
| Higher | 3,784 | 9.8 | 3467 | 18.4 | 2332 | 25.4 | 3929 | 34.5 |
| **Religion** |  |  |  |  |  |  |  |  |
| Hindu | 32,260 | 83.4 | 14504 | 77.1 | 6769 | 73.7 | 8256 | 72.5 |
| Muslim | 5,087 | 13.1 | 3141 | 16.7 | 2025 | 22.1 | 2609 | 22.9 |
| Christian | 736 | 1.9 | 449 | 2.4 | 229 | 2.5 | 220 | 1.9 |
| Others | 620 | 1.6 | 715 | 3.8 | 158 | 1.7 | 297 | 2.6 |
| **Social group** |  |  |  |  |  |  |  |  |
| GEN | 4,692 | 12.1 | 3751 | 19.9 | 2107 | 23.0 | 3212 | 28.2 |
| SC | 9,603 | 24.8 | 4218 | 22.4 | 2148 | 23.4 | 1981 | 17.4 |
| ST | 5,719 | 14.8 | 1597 | 8.5 | 453 | 4.9 | 458 | 4.0 |
| OBC | 16,728 | 43.2 | 8091 | 43.0 | 4019 | 43.8 | 4917 | 43.2 |
| Don't know | 1,962 | 5.1 | 1152 | 6.1 | 454 | 5.0 | 813 | 7.1 |
| **Household wealth quintile** |  |  |  |  |  |  |  |  |
| Poorest | 14,652 | 37.9 | 3063 | 16.3 | 687 | 7.5 | 176 | 1.6 |
| Poorer | 10,637 | 27.5 | 4123 | 21.9 | 1198 | 13.1 | 624 | 5.5 |
| Middle | 7,475 | 19.3 | 4505 | 24.0 | 1925 | 21.0 | 1553 | 13.7 |
| Richer | 4,376 | 11.3 | 4359 | 23.2 | 2549 | 27.8 | 3350 | 29.4 |
| Richest | 1,564 | 4.0 | 2758 | 14.7 | 2822 | 30.7 | 5678 | 49.9 |
| **Mass media exposure** |  |  |  |  |  |  |  |  |
| No | 15,151 | 39.2 | 4588 | 24.4 | 1422 | 15.5 | 1047 | 9.2 |
| Partial | 21,974 | 56.8 | 12704 | 67.5 | 6951 | 75.7 | 9090 | 79.9 |
| High | 1,579 | 4.1 | 1517 | 8.1 | 808 | 8.8 | 1244 | 10.9 |
| **Water facility on household premises** |  |  |  |  |  |  |  |  |
| Yes | 25,643 | 66.3 | 14285 | 76.0 | 7230 | 78.8 | 9655 | 84.8 |
| No | 13,061 | 33.8 | 4523 | 24.1 | 1951 | 21.3 | 1726 | 15.2 |
| **Household sanitation facility** |  |  |  |  |  |  |  |  |
| Improved | 23,101 | 60.2 | 15974 | 85.6 | 7596 | 83.2 | 10909 | 96.0 |
| Unimproved | 937 | 2.4 | 387 | 2.1 | 270 | 3.0 | 209 | 1.8 |
| Open defecation | 14,328 | 37.4 | 2298 | 12.3 | 1261 | 13.8 | 243 | 2.1 |
| **Region** |  |  |  |  |  |  |  |  |
| North | 3,648 | 9.4 | 3492 | 18.6 | 1330 | 14.5 | 1973 | 17.3 |
| Central | 11,962 | 30.9 | 5230 | 27.8 | 2033 | 22.2 | 2423 | 21.3 |
| East | 12,883 | 33.3 | 4117 | 21.9 | 1885 | 20.5 | 1537 | 13.5 |
| Northeast | 1,870 | 4.8 | 660 | 3.5 | 264 | 2.9 | 178 | 1.6 |
| West | 3,508 | 9.1 | 2325 | 12.4 | 1427 | 15.5 | 2443 | 21.5 |
| South | 4,832 | 12.5 | 2985 | 15.9 | 2242 | 24.4 | 2827 | 24.8 |

Supplementary Table 3: Summary result of Fairlie decomposition analysis showing the mean difference in the unsafe disposal of children stools between rural and urban India, 2019-21

| Total number of observations | 79,618 |
| --- | --- |
| Total number of observation (rural) | 63621 |
| Total number of observation (urban) | 15997 |
| Mean prediction for rural | 0.66346 |
| Mean prediction for urban | 0.45477 |
| Mean difference (rural-urban) | 0.20869 |
| Total explained | 0.16601 |
| Percentage explained | 79.55 |
| Percentage unexplained | 20.45 |
